# High-power, electrically-driven continuous-wave 1.55-μm Si-based multi-quantum well lasers with a wide operating temperature range grown on wafer-scale InP-on-Si (100) heterogeneous substrate

**DOI:** 10.1038/s41377-024-01389-2

**Published:** 2024-03-11

**Authors:** Jialiang Sun, Jiajie Lin, Min Zhou, Jianjun Zhang, Huiyun Liu, Tiangui You, Xin Ou

**Affiliations:** 1 National Key Laboratory of Materials for Integrated Circuits, Shanghai Institute of Microsystem and Information Technology, CAS, Shanghai, 200050 China; 2 Center of Materials Science and Optoelectronics Engineering, University of Chinese Academy of Sciences, 100049 Beijing, China; 3 College of Information Science and Engineering, Jiaxing University, Jiaxing, 314001 China; 4 Beijing National Laboratory for Condensed Matter Physics, Institute of Physics, Chinese Academy of Sciences, 100190 Beijing, China; 5 Department of Electronic and Electrical Engineering, University College London, London, WC1E 7JE UK

**Keywords:** Diode lasers, Photonic devices

## Abstract

A reliable, efficient and electrically-pumped Si-based laser is considered as the main challenge to achieve the integration of all key building blocks with silicon photonics. Despite the impressive advances that have been made in developing 1.3-μm Si-based quantum dot (QD) lasers, extending the wavelength window to the widely used 1.55-μm telecommunication region remains difficult. In this study, we develop a novel photonic integration method of epitaxial growth of III-V on a wafer-scale InP-on-Si (100) (InPOS) heterogeneous substrate fabricated by the ion-cutting technique to realize integrated lasers on Si substrate. This ion-cutting plus epitaxial growth approach decouples the correlated root causes of many detrimental dislocations during heteroepitaxial growth, namely lattice and domain mismatches. Using this approach, we achieved state-of-the-art performance of the electrically-pumped, continuous-wave (CW) 1.55-µm Si-based laser with a room-temperature threshold current density of 0.65 kA/cm^−2^, and output power exceeding 155 mW per facet without facet coating in CW mode. CW lasing at 120 °C and pulsed lasing at over 130 °C were achieved. This generic approach is also applied to other material systems to provide better performance and more functionalities for photonics and microelectronics.

## Introduction

As a result of the increasingly fast-growing data traffic in current high-performance computing systems and data centers, issues associated with power consumption, transmission speed and bandwidth have been severely exacerbated by the limitations of conventional electrical interconnections. In recent years, silicon photonics has attracted tremendous attention as one of the most promising photonic solutions for future optical communications by leveraging the mature and cost-effective complementary metal–oxide–semiconductor (CMOS) process^1–5^. However, a reliable, efficient electrically-driven integrated light-emitting device has long been considered to be the key missing building block in Si-based photonics integrated circuits (PICs) due to the indirect band gap of Si, which leads to the requirement of additional momentum for the light emission process^6^. Monolithic integration of III-V compound semiconductors with excellent optoelectronic properties and Si-based substrates is one of the most promising candidates for reliable on-chip laser applications^7^. Due to the advantages of quantum dots (QDs), such as high tolerance to crystalline defects and high temperature stability, impressive progress has been made in developing GaAs-based lasers on Si substrate emitting at 1.3 μm with low threshold current and excellent lifetime achieved by heteroepitaxial growth^1,8–10^. However, due to the high-density dislocations generated as a result of the large lattice mismatch between InP and Si (8%), it is extremely difficult to extend the operating wavelength to the more comprehensive 1.55 μm C-band based on the InP platform, which is crucial for applications in the fields of low-loss transmission in mid-/long-haul communication, sensing, and light detection and ranging (LiDAR)^11–13^.

The initial attempts to achieve a 1.55-μm wavelength integrated light source on a Si substrate date back to the 1990s. The most straightforward approach involved depositing InP thin films on Si with a 2-μm GaAs layer as an intermediate layer to alleviate the large lattice mismatch that generates high-density threading dislocations (TDs)^14,15^. Despite achieving impressively low threading dislocation density (TDD) and excellent lasers’ lifetime, the aggressively thick (up to 15 μm) III–V buffer layers may result in severe thermal cracks and cannot efficiently provide evanescent coupling to the Si waveguide underneath^16,17^. More recently, an InGaAs/InAlGaAs multi-quantum well (MQW) laser with a much thinner (1.5 μm) InP buffer was grown on an InP-on-V-grooved Si(100) substrate, while the room-temperature (RT) lasing was only achieved under pulsed operation^18^. In 2019, Shi et al. reported RT continuous-wave (CW) lasing with a Fabry–Perot (FP) laser by using a InGaAsP MQW active layer as the active gain medium on an InP-on-V-grooved Si (100) substrate^19^. The FP lasers have a threshold current density of 2.05 kA/cm^2^ at RT, and can be operated at up to 65 °C under CW mode with a relatively thin III-V buffer layer (5.9 μm). In contrast, a rapid device failure occurred at 60 °C after just 5.6 hours of aging under pulsed operation due to the high TDD of 1.15 ×10^8 ^cm^−3^ ^20^. Besides InGaAs/InGaAsP MQW lasers, InAs/InAlGaAs quantum dashes (QDashs) and QDs have also been used as the active gain medium emitting at the 1.55 μm telecommunication wavelength. Using a quantum-dash-in-well structure as the active medium, Xue et al. demonstrated a FP laser with a threshold current density of 1.3 kA/cm^2^ at RT, which emitted at up to 59 °C under CW operation^21^. Taking advantage of the use of nanopatterned V-grooved Si(100) substrates and InGaAs/InP dislocation filters, Zhu et al. reported a FP laser with a threshold current density of 1.6 kA/cm^2^ at RT, which emitted at up to 80 °C, but with pulsed operation only^22^.

In general, the crystalline quality of III-V directly grown on Si substrate is still unsatisfactory with a TDD of up to 10^8 ^cm^−2^ due to the large lattice mismatch, the difference in thermal expansion coefficients, and various polarities between III-V and Si. To develop high-quality and reliable on-chip semiconductor thin film applications, great efforts have been devoted to the fabrication and transfer of surface thin films, e.g., thin film lithium niobate integrated platform fabricated by electron-beam lithography (EBL) for electro-optic modulators^23^, GaN thin film fabricated by laser lift-off (LLO) for flexible device applications^24^, free-standing h-BN film synthesized by solution-based method for nonlinear optics^25^, etc. Previously, we reported the wafer-scale InP-on-Insulator (InPOI) heterogeneous integration substrate fabricated by exfoliating an InP thin film from the InP bulk wafer and transferring it onto a Si-based handle wafer using the ion-cutting technique^26^. The InPOS/InPOI substrate can be used as a template for the epitaxial growth of III-V to realize heterogeneous lasers on Si, which decouples the correlated root causes of the detrimental dislocations during heteroepitaxy, namely lattice and domain mismatches.

In this study, an approximately 2-μm-thick AlGaInAs MQW laser structure was epitaxially grown on a wafer-scale InPOS heterogeneous substrate fabricated by the ion-cutting technique. The fabricated FP lasers emitted at 1.54 μm operating at 120°C under CW mode and at over 130 °C under pulsed mode, with a threshold current density of 0.65 kA/cm^−2^ and output power exceeding 155 mW/facet at RT without any facet coating. The performance data from the laser proof-of-concept study suggest that this ion-cutting plus epitaxial growth approach is one of the most promising solutions to achieve a C-band light source integrated on a CMOS-compatible Si (100) substrate for high volume manufacting. In principle, it is also applicable for the integration of many more different materials.

## Results

### Substrate fabrication and material quality characterizations

**Fig. 1:**
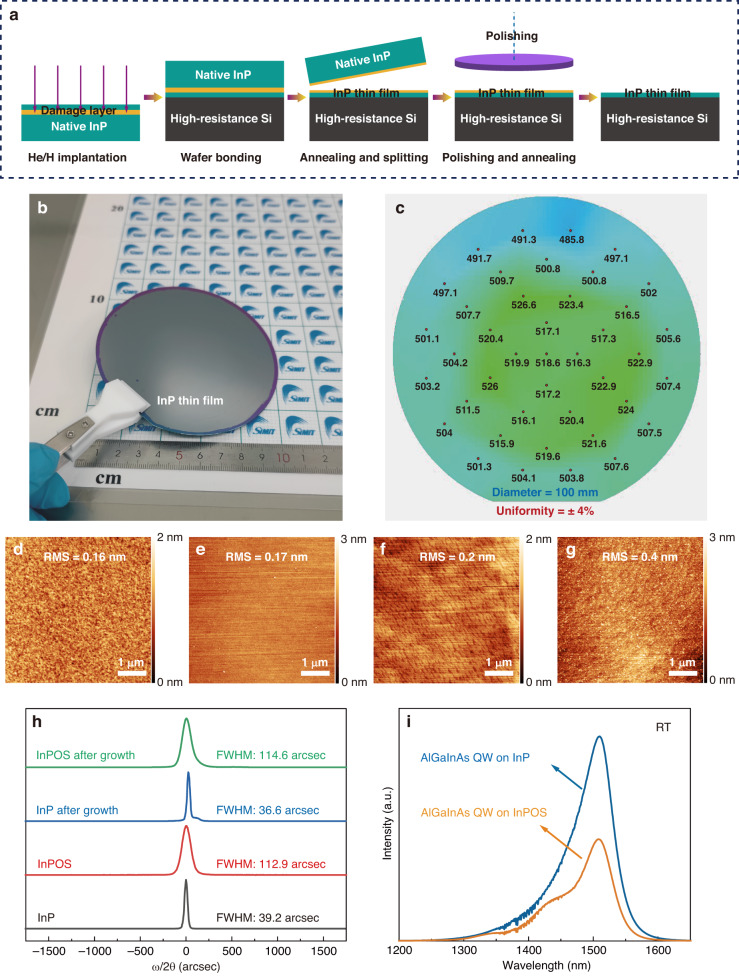
Fabrication of InP-on-Si (100) substrate and material characterization before and after epitaxy. **a** The schematic diagram of the fabrication process of InPOS by ion-cutting technique. **b** Image of 4-inch as-transferred InP thin film on a Si-based substrate by ion-cutting. **c** Thickness mapping of the polished InP thin film. The AFM images of (**d**) bulk InP, (**e**) InPOS, (**f**) bulk InP after MOCVD growth and (**g**) InPOS after MOCVD growth. **h** The normalized (004) X-ray rocking curves of the bulk InP and InPOS before and after MOCVD epitaxial growth (**i**) RT-PL spectra of the AlGaInAs MQWs structure grown on the bulk InP and InPOS

The process of integrating InP on Si (001) substrates by the ion-cutting technique is illustrated in Fig. 1a. A sequential co-implantation of He and H ions was initially carried out on a 2-inch bulk InP wafer at room temperature, and the ion energy/fluence of the He and H ion implantation were 115 keV/2 ×10^16 ^cm^−2^ and 75 keV/5 ×10^16 ^cm^−2^, respectively. Then, a surface activated bonding (SAB) technique was used to bond the implanted InP wafer with a 4-inch high-resistance Si wafer at room temperature. Both the implanted InP surface and the polished surface of high-resistance Si were first activated by an Ar fast atom beam (FAB) for 60 s with a power source of 1 keV and 75 mA using a WAP-100T bonding system. Then the activated wafers were bonded at the vacuum pressure of 1 ×10^−5 ^Pa with a bonding pressure of about 5 MPa. Subsequently, a thin layer of monocrystalline InP was transferred onto the high-resistance Si wafer after an annealing at 200 °C for 2 h under N_2_ atmosphere. This is different from previous works using hydrophilic wafer bonding^26^, as there was no accessory gas generated at the bonding interface when using the hydrophobic SAB technique, a Si wafer without the outgassing channels was used in this work. Chemical mechanical polishing (CMP) was ultimately used to remove the damage layer introduced by ion implantation and smooth the surface for epitaxial growth. A typical 4-inch as-transferred InP-on-insulator (InPOI) substrate fabricated by our group is shown in Fig. 1b, and has an excellent non-uniformity of ± 4% over the entire wafer as shown in Fig.1c.

An AlGaInAs MQW laser structure was then grown on InPOS using an Aixtron MOCVD system. For comparison, the same structure was also grown on a commercial bulk n^+^-InP wafer under the same growth conditions. It is important to note that the MOCVD growth process was standardized for the bulk InP substrate which had not been optimized for InPOS, considering the different thermal conductivity and thermal expansivity between Si and InP. The crystalline quality and surface roughness of the epitaxial layers have a major impact on the performance of Si-based lasers. The noticeable thickness variations in the MQWs lead to severe carrier traps at the interfaces between wells and barriers^27,28^. These traps and crystalline defects, which serve as sources of current leakage and non-radiative recombination centers, deteriorate the laser performance. The surface morphologies of bulk InP and InPOS before the epitaxial growth were characterized by atomic force microscopy (AFM) in a 5 μm × 5 μm scanning area as shown in Fig. 1d, e. The as-prepared InPOS shows high-quality surface finish with only 0.17 nm surface roughness, which is comparable to that of the bulk InP (0.16 nm). After a MOCVD growth process standardized for the bulk InP substrate, the root-mean-square (RMS) roughness value on the InPOS (0.4 nm) increased slightly above that on the bulk InP (0.2 nm), as shown in Fig. 1f, g. It is expected that the epitaxial layers with lower RMS roughness value can be achieved under well optimized epitaxial growth conditions for the InPOS heterogeneous substrate. However, the InPOS surface after epitaxial growth with an RMS roughness value of 0.4 nm is smooth enough to facilitate the device fabrication without any pinholes, which is essential for high-performance lasing^22^.

The crystalline qualities of both bulk InP and InPOS before and after epitaxial growth were characterized by analyzing X-ray diffraction (XRD) rocking curves along the InP (004) reflection, as shown in Fig. 1h. The full width at half maximum (FWHM) of the InP layer on the InPOS is 112.9 arcsec, which is 2.88 times wider than that of the bulk InP (39.2 arcsec). This was due to the residual strain induced during the ion-cutting process. After the epitaxial growth, the epitaxial layers on the bulk InP show a narrowing of the FWHM (from 39.2 to 36.6 arcsec). However, a slight increase of the FWHM (from 112.9 to 114.6 arcsec) was found on the InPOS under the same growth conditions, which might be due to defect propagation and evolution during the MOCVD process. To further evaluate the optical quality of MQW structures, RT photoluminescence (PL) measurements were performed on both bulk InP and InPOS without cladding and contact layers, excited by a CW laser at a wavelength of 980 nm and a power of 60 mW. As shown in Fig. 1i, the PL peak wavelength for the QWs on the InPOS is centered at 1507.8 nm, while that for the bulk InP is at 1509.6 nm. The slight variation in the PL peak wavelength may be due to residual strain in the MQWs of the InPOS. The PL peak intensity of MQWs on InPOS reaches 50% of that on the bulk InP, with comparable FWHM values of 67.5 and 70.0 meV for InPOS and bulk InP, respectively. We believe the AlInGaAs MQW design still has room to improve by optimizing the FWHM. In general, the InPOS heterogeneous substrate fabricated by the ion-cutting technique with high crystalline quality and low surface roughness is adequate for the fabrication of high-performance lasers on Si substrate.

**Fig. 2:**
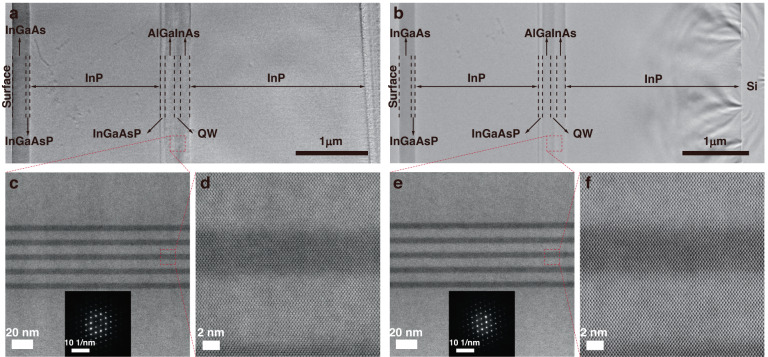
TEM characterization of the epitaxial structure grown on InP and InPOS. Cross-sectional TEM images of AlGaInAs MQW laser structures on (**a**) bulk InP and (**b**) InPOS. STEM images of entire MQW structures on (**c**) bulk InP and (**e**) InPOS. The amplified QW structures on (**d**) bulk InP and (**f**) InPOS

The microstructures of epitaxial layers grown on the bulk InP and InPOS were characterized using transmission electron microscopy (TEM) and scanning TEM (STEM). Cross-sectional TEM images of the overall epitaxial structures on the bulk InP and InPOS are shown in Fig. 2a, b, respectively. These TEM images reveal that on both bulk InP and InPOS, the resolved multilayer interfaces can be clearly distinguished without obvious threading dislocations in the epitaxial layers. It is worth noting that the interface between the transferred InP thin film by the ion-cutting technique and the epitaxial InP contact layer are not visible in Fig. 2b, indicating the excellent crystalline-quality of InPOS. The active regions, the most crucial components of the entire laser system, were examined by STEM, as shown in Fig. 2c–f. The interfaces between the Al_0.24_GaIn_0.71_As well layers and Al_0.44_GaIn_0.49_As barrier layers are well distinguished by the different contrast of atoms, and the atoms of the active regions are arranged in a regular lattice structure without any visible dislocations on both the bulk InP and InPOS. Selected-area electron diffraction (SAED), at the atomic-scale resolution, was used to further evaluate the crystalline quality of the active regions. As shown in the insets in Fig. 2c, e, the SAED images of the active regions show regular and bright spots rather than diffraction rings, which suggests that the active regions have high single-crystalline quality on both the bulk InP and InPOS. Thus, this suggested that the crystalline quality of the epitaxial layers on the InPOS are comparable to that on the bulk InP, without the obvious misfit and threading dislocations that are usually introduced during heteroepitaxial growth^29–31^.

### Laser performance characteristics

**Fig. 3:**
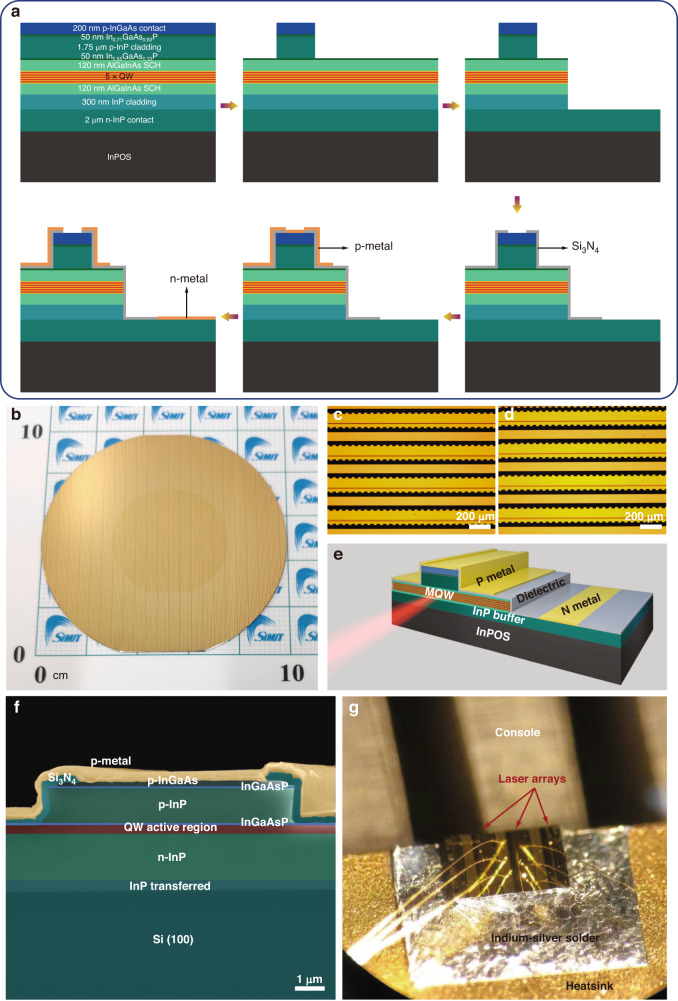
Fabrication and package of laser based on InPOS. **a** Schematic diagram of the laser fabrication process. **b** wafer-scale patterned laser devices based on InPOS substrate. Optical microscopy images of as-fabricated laser bars on the bulk InP (**c**) and InPOS (**d**). **e** 3D schematic diagram of AlGaInAs MQW laser on InPOS. **f** Titled cross-section false color SEM image of an as-cleaved 8 μm × 1500 μm laser on InPOS. **g** As-cleaved and packaged laser arrays

Ridge lasers with cavity lengths ranging from 750 to 1750 µm were fabricated using a “top-top” contact scheme as schematically depicted in Fig. 3a. Patterned laser devices, based on wafer-sale InPOS substrate, were successfully fabricated as shown in Fig. 3b. The surface morphology of the devices fabricated on the bulk InP and InPOS prior to cleaving are shown in Fig. 3c, d, respectively. No obvious defects reaching to the surface were observed. A three-dimensional (3D) schematic diagram of the Si-based laser and is shown in Fig. 3e. The false color scanning electron microscopy (SEM) images of an as-prepared FP laser (8 μm ridge width) on InPOS is displayed in Fig. 3f. The laser arrays to be measured were ultimately cleaved and packaged as shown in Fig. 3g.

**Fig. 4:**
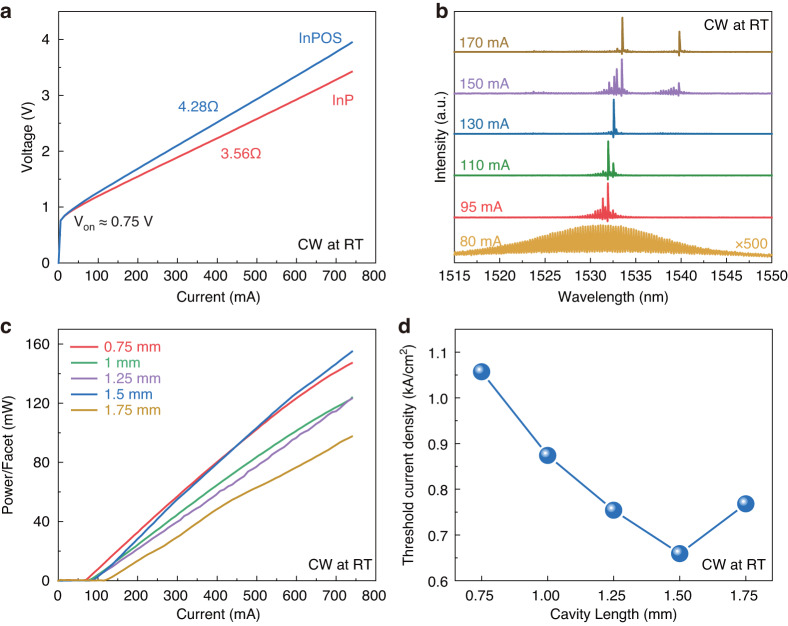
Performance of lasers on InPOS with increasing current and different cavity lengths. **a** Typical I-V characteristics of as-cleaved lasers on the bulk InP and InPOS with cavity length of 1500 μm and ridge width of 8 μm. **b** Lasing spectra for an 8 μm × 1500 μm laser on InPOS at various injection currents. **c** L-I characteristics for the laser on InPOS for various cavity lengths under CW operating conditions. **d** The relationship between the J_th_ and cavity length for the laser on InPOS

The current-voltage (I–V) characteristics at 20 °C for the 8 µm × 1500 µm ridge lasers fabricated on the bulk InP and InPOS are shown in Fig. 4a. The lasers on both the bulk InP and InPOS have nearly equal turn voltage of about 0.75 V, while the extracted series resistance on the InPOS (4.28 Ω) is slightly higher than that on the bulk InP (3.56 Ω). Compared to the thick InP layer (more than 100 μm) remaining after lapping for lasers on the bulk InP, the n-InP contact layer for lasers on the InPOS is significantly thinner (2 μm), which contributes extra lateral resistance to the total series resistance. The lasing spectra of an 8 μm × 1500 μm laser on the InPOS at different injection currents under CW mode are shown in Fig. 4b. At a relatively low driving current of 80 mA, a broad spontaneous emission spectrum with a peak wavelength at around 1533 nm and an FWHM of 16.5 nm was observed. With progressively higher injected currents, the transition from spontaneous emission to stimulated emission with numerous longitudinal modes occurred. Additionally, a slight red-shift of the lasing wavelength with increasing injection current was also observed, which could be mainly attributed to the self-heating effect. The lasing properties of lasers on the InPOS for various cavity lengths, measured under CW operating conditions at RT (20 °C), are shown in Fig. 4c, d. With the increase of the cavity length from 750 to 1750 µm, the threshold current increases due to the increased current injection area, as shown in Fig. 4c, while the threshold current density (J_th_) decreases, as shown in Fig. 4d. It is important to note that the uneven increased J_th_ for the 1750 µm long laser may be due to the improperly split facet. The minimum J_th_ of 0.65 kA/cm^−2^ was achieved with a cavity length of 1.5 mm at RT under CW mode, which is the lowest J_th_ ever recorded for a Si-based FP laser emitting at 1.55 μm^20^.

**Fig. 5:**
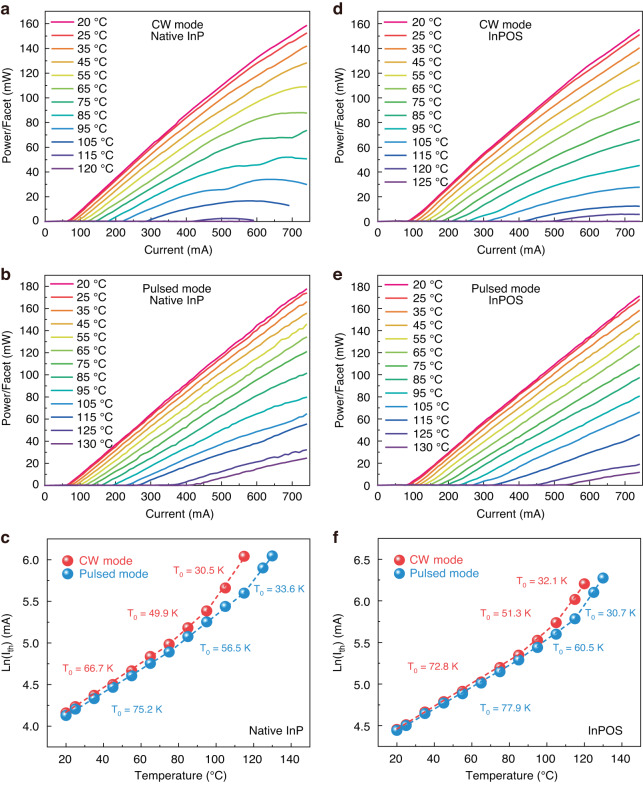
Performance of lasers on InP and InPOI at different temperatures. L-I characteristics for the 8 µm×1500 μm laser on the bulk InP under (**a**) CW and (**b**) pulsed modes at different temperatures. **c** Temperature dependence of the threshold current for laser on the bulk InP. L-I characteristics for the 8 µm × 1500 μm laser on the InPOS under (**d**) CW and (**e**) pulsed modes at different temperatures. **f** Temperature dependence of the threshold current for laser on the InPOS

For datacom application, lasers are required to operate at high temperatures with sufficient output power under CW conditions. In order to evaluate the performance of our devices at high temperature, the temperature-dependent light output *versus* current (L-I) characteristics were measured under CW and pulsed modes for the lasers on both the bulk InP and InPOS with identical ridge width and cavity length (8 μm/1500 μm) as shown in Fig. 5. A thermoelectric cooler (TEC) system was used to control the stage temperature. As a result of the excellent crystalline quality of the MQWs structure grown on InPOS, the single-facet output power for the laser on the InPOS was as high as 155 mW at an injection current of 740 mA under CW operation at RT, and was not even saturated at the higher injection current. The single-facet output power of 155 mW on the InPOS is comparable to that (164 mW) of the laser on the bulk InP, which is much higher than the maximum single-facet output power of 22 mW for a 1.55 μm Si-based FP laser operating under CW mode at RT, as previously reported^21^. The laser on the bulk InP reached its maximum operational temperature at 115 °C under CW operation. It is impressive that the laser on the InPOS can operate at a higher temperature of 120 °C. To the best of our knowledge, 120 °C is the highest operating temperature achieved to date for the Si-based FP laser emitting at 1.55 μm under CW mode. It is believed that the maximum operating temperature in lasers grown on Si is typically limited by the growth dislocation /defect density under working conditions due to the recombination-enhanced climb process^10,32^. The high-temperature operation up to 120 °C under CW mode for the lasers on the InPOS can be attributed to the low defect density as shown in Fig. 2, which results in negligible defect evolution and penetration in the active region.

The pulsed operation with a duty cycle of 0.5% and a pulse width of 2 μs was performed to avoid the self-heating effect. Under pulsed mode, the lasers on both bulk InP and InPOS could be operated at above 130 °C, which was limited by the temperature range of the TEC controller, as shown in Fig. 5b, e. The threshold currents at RT for lasers on the bulk InP and InPOS are 65 mA/60 mA and 86 mA/82 mA under CW and pulsed operations, respectively. Compared to the case of CW mode, there was no significant reduction in threshold current for the lasers on the bulk InP and InPOS under pulsed mode at a low temperature range, indicating that the device self-heating at low temperature did not dominate the performance of thermal stability. However, at the high temperature of 115 °C, there was a significant reduction of threshold current for the lasers under pulsed operation compared to those under CW operation, which were 270 mA/420 mA and 325 mA/410 mA for the lasers on the bulk InP and InPOS, respectively. This indicated that the effect of self-heating was enhanced at high temperatures. The lasers on the InPOS show a threshold current reduction rate of 20.7% between the CW and pulsed modes, which is smaller than that of lasers on the bulk InP (35.7%). Due to the higher thermal conductivity of Si (145 W/m·K) than that of InP (68 W/m·K)^33^, the heat of the lasers on the InPOS can dissipate more quickly, and thus self-heating has less impact on the threshold current.

The average characteristic temperature T_0_, which is an important measure of the temperature sensitivity of the semiconductor laser diodes, can be calculated using the following equation^34^:

1 $${I}_{{th}}\left(T\right)={I}_{0}\times \exp \left(\frac{T}{{T}_{0}}\right)$$

where I_th_ is the threshold current at different temperatures, I_0_ is threshold current at RT, T is the operating temperature. The threshold densities at various temperatures are shown in Fig. 5c, f for the lasers on the bulk InP and InPOS, respectively. The characteristic temperature T_0_ was obtained in different temperature ranges. For the lasers on the InPOS under CW operation, the calculated characteristic temperature T_0_ was 72.8 K between 20 and 85 °C, 51.3 K between 85 and 105 °C, and 32.1 K between 105 and 120 °C. Under the pulsed mode with a duty cycle of 0.5%, the T_0_ value was higher (77.9 K from 20 to 85 °C and 60.5 K from 85 to 115 °C) than that under CW mode in the temperature range of 20 to 115°C. However, it is the reverse between 115 and 130 °C, probably due to the different temperature range used for the calculation of T_0_. The T_0_ values of the lasers on the bulk InP were 66.7 K between 20 and 75 °C, 49.9 K between 75 and 95 °C, and 30.5 K between 95 and 115°C under CW operations, which are lower than those on the InPOS. In addition, the temperature stability under pulsed mode was also not improved for the lasers on the bulk InP (75.2 K between 20 and 75 °C, 56.5 K between 75 and 105°C, and 33.6 K between 105 and 130 °C). It is widely believed that the T_0_ of the AlGaInAs/InP laser system is dominated by the Auger recombination process and the leakage current^35,36^. The leakage current acts as the primary factor for temperature sensitivity, which is an inevitable mechanism under high temperature operation. Thus, the T_0_ value of a laser grown on Si by hetero-epitaxial growth is generally lower than that grown on bulk InP with identical device construction due to the high leakage current caused by the high TDD. However, as a result of the high-quality crystal InP film of the InPOS in this study, few defects acting as non-radiative recombination centers and current leakage sources were introduced in the active region. Combined with the higher thermal conductivity of Si, the better temperature characteristics was achieved for the lasers on the InPOS.

**Fig. 6:**
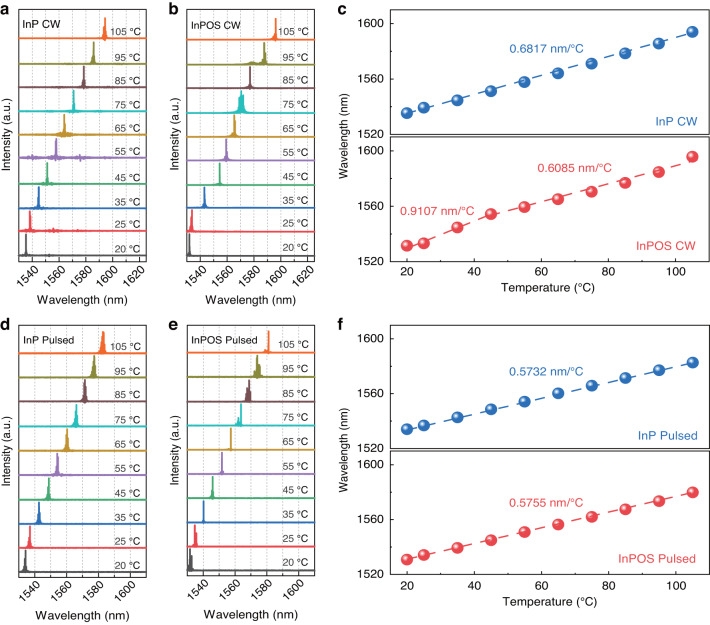
Characterization of wavelength shift for lasers on InP and InPOS with increasing temperature. Lasing spectra for 8 µm × 1500 μm lasers on (**a**) bulk InP and (**b**) InPOS under CW mode at different temperatures. **c** Temperature dependence of the lasing wavelength for lasers on the bulk InP and InPOS under CW mode. Lasing spectra for 8 µm × 1500 μm lasers on (**d**) bulk InP and (**e**) InPOS under pulsed mode at different temperatures. **f** Temperature dependence of the lasing wavelength for lasers on the bulk InP and InPOS under pulsed mode

The lasing spectra of lasers on the bulk InP and InPOS driven by 1.2 times threshold current at various operating temperatures under CW mode are presented in Fig. 6a–b. At 20 °C, the peak wavelengths of lasers on the bulk InP and InPOS are located at 1535 nm and 1531 nm, respectively. The difference of the lasing wavelengths was likely attributed to the residual thermal strain in the MQWs of the InPOS^37^. In addition, the composition changes caused by the growth temperature variation, due to the different thermal conductivity of Si and InP, also accounted for the peak wavelength change. With increasing operating temperature^38^, the band gap of the active area narrows, resulting in a redshift towards longer emission wavelengths. Moreover, the self-heating effect caused by the increasing injection currents should also account for the wavelength redshift^39,40^. The average red-shift rate (Δλ/ΔT) of the laser on the bulk InP was 0.6817 nm/°C from 20 to 105 °C under CW mode, as shown in Fig. 6c. However, for the laser on the InPOS, two different red-shift rates occurred in the low temperature range (20–45 °C) and high temperature range (45–105 °C). In the low temperature range, the self-heating effect is more severe than that of lasers on InP due to the larger threshold current under the 1.2-times-threshold test condition, resulting in a larger red-shift rate of 0.9107 nm/°C. With further increase of the operating temperature, the advantage of the high thermal conductivity of Si becomes dominant, thereby reducing the thermal accumulation at the active area. Therefore, the self-heating effect was significantly reduced, resulting in a lower redshift rate of 0.6085 nm/°C in the high temperature range. To further confirm the impact of the self-heating effect under CW mode, the lasing spectra of lasers on the bulk InP and InPOS driven by 1.2 times threshold current at various operating temperatures under pulsed mode were measured as shown in Fig. 6d, e. At 20 °C, the peak wavelengths of lasers on the bulk InP and InPOS under pulsed mode shift towards a short wavelength compared to those in CW mode, i.e., the peak wavelength shifts from 1535 to 1533 nm and 1531 to 1530 nm for the lasers on the bulk InP and InPOS, respectively, as a result of the negligible self-heating effect under pulsed mode. As shown in Fig. 6f, lower average red-shift rates of 0.5732 and 0.5755 nm/°C were achieved under pulsed mode for lasers on bulk InP and InPOS, respectively. The similar red-shift rates for the lasers on bulk InP and InPOS also suggest that the self-heating effect could be ignored under pulsed mode and that the quality of ion-cutting InP thin film is comparable to that of the InP bulk. In general, the redshift rate of lasers on InPOS is less than that of QW lasers lasing at 1.55 μm directly grown on Si have reported^19,41^.

**Fig. 7:**
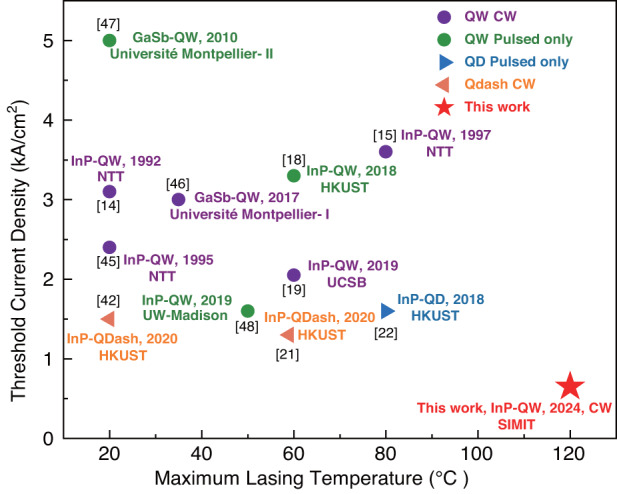
The historical evolution of 1.55-μm band lasers monolithically integrated on Si by direct epitaxial growth in terms of threshold current density reduction and increase in maximum lasing temperature^14,15,18,19,21,22,42,45–48^

The improvement in threshold current density reduction and the maximum lasing temperature of Si-based FP lasers emitting at 1.55 μm integrated through direct growth are shown in Fig. 7. Since 1992, many efforts have been made in the design of the laser structure including buffer layers, active region, as well as TDD suppression by optimizing the template geometrical morphology^18,19,42^. After 2015, numerous efforts have been devoted to developing the monolithic integration of lasers with Si-based CMOS platform. To the best of our knowledge, we have achieved the lowest reported threshold current density (0.65 kA/cm^−2^) compared to those electrically-driven Si-based lasers emitting at 1.55 μm by direct epitaxial growth, and the device has threshold that is comparable to the best result of Si-based lasers emitting at 1.55 μm integrated by other methods (600 A/cm^−2^)^43^, which can operate at the highest temperature (120 °C). Future efforts will be focused on lowering the J_th_ and improving the temperature characteristics.

## Discussion

Based on a novel wafer-scale InPOS heterogeneous substrate obtained by the ion-cutting technique, we have developed RT CW lasing of electrically-driven AlGaInAs MQW lasers emitting at 1.55 μm with outstanding performance. The buffer thickness was only 2 μm and can be even thinner in our scheme, which is highly essential for efficient light coupling for practical applications. This study also demonstrated that the ion-cutting plus epitaxy approach is promising and cost-competitive to combine wafer-scale dissimilar materials by decoupling the correlated root causes, i.e., lattice and domain mismatches. Overall, the high-quality QW active region and smooth surface morphology contribute to the excellent laser performance achieved at a low threshold current density of 0.65 kA/cm^−2^, an output power exceeding 155 mW per facet, and an operating temperature up to 120 °C under CW mode. Compared to the MQW lasers on the bulk InP, the MQW lasers on the InPOS showed superior thermal characteristics as indicated by the temperature-dependent L-I measurements and lasing spectra. In the next step, it is important to consider the compatibility with the mature SOI platform and interconnect these high-performance MQW lasers with other photonic components, e.g., waveguide, modulators and photodetectors, for the advanced all-in-one photonic integration. We believe that this ion-cutting plus epitaxy strategy will pave the way for large-density and highly-scalable silicon photonic integration and opens up new possibilities for full photonics integration on the mature Si-based CMOS microelectronics system.

## Materials and methods

As previously reported^26,44^, the InPOS was first fabricated using the ion-cutting technique, which is illustrated in Fig. 1a The AlGaInAs MQW laser structure was then grown on the InPOS using an Aixtron® metalorganic chemical vapor deposition (MOCVD) system (Aixtron SE, Herzogenrath, Germany). Prior to epitaxial growth, in situ deoxidation was performed on the InPOS in the MOCVD chamber. A 2-μm Si-doped n-InP buffer layer, used as a contact layer, was initially grown on the epi-ready InPOS followed by a 300-nm Si-doped n-InP cladding layer with doping concentrations of 3 × 10^18^ and 1 × 10^18 ^cm^−3^, respectively. Afterwards, the active region containing five layers of 6 nm Al_0.24_GaIn_0.71_As wells and 10 nm Al_0.44_GaIn_0.49_As barriers sandwiched between 120-nm-thick AlGaInAs separate confinement heterostructures (SCHs) were grown. Then, a 50-nm In_0.85_GaAs_0.33_P barrier layer facilitating the ridge waveguide fabrication with a doping concentration of 1 × 10^18 ^cm^−3^ was grown. Finally, a 1750-nm-thick Zn-doped InP p-cladding layer and a 200-nm-thick In_0.53_GaAs p-contact layer were deposited with a 50-nm-thick In_0.71_GaAs_0.62_P intermediate layer to enhance the transition of carriers and reduce the operating voltage. For reference, a commercial bulk n^+^-InP was also send into the MOCVD chamber to grow the same structure but without the 2-μm InP buffer layer.

After the MOCVD growth, the ridge devices with cavity lengths ranging from 750 to 1750 µm were fabricated using standard photolithography followed by a wet etching process. Due to the high-resistance Si substrate in the InPOS, a “top-top” contact scheme was used. The top mesa was selectively wet-etched to form the ridge waveguide that stopped just above the 50-nm-thick InGaAsP barrier layer to avoid damaging the MQWs. The necessary exposure of the n-contact layer was formed by selectively wet etching through the whole structure down to the InP buffer layer. This was followed by sidewall passivation with a 300-nm-thick Si_3_N_4_ layer deposited for electrical isolation by plasma-enhanced chemical vapor deposition (PECVD), and using reactive ion etching (RIE) to expose the contact windows. Ti/Pt/Au and Ge/Au/Ni/Au were evaporated by a lift-off process for p- and n-contact, respectively. After rapid thermal annealing and substrates thinning, the wafer was cleaved into laser bars without any facet coating.

The as-cleaved laser bars were positioned on a gold-plated ceramic heatsink using an indium–silver low-melting-point solder and a bonded gold wire to facilitate testing. Then, after the heatsink was mounted on a copper stage with a TEC system (Thorlabs Inc., Newton, NJ, USA), the light output from the waveguide facet was collected by a Thorlabs photodetector and characterized by an optical spectral analyzer (Thorlabs Inc.).
